# 2,2′-(*p*-Phenyl­enedimethyl­ene)bis­(propane-1,3-diol)

**DOI:** 10.1107/S1600536808041688

**Published:** 2008-12-20

**Authors:** Yajun Gao, Haitao Xi, Xiaoqiang Sun, Yongsheng Fu, Li Liu

**Affiliations:** a School of Chemistry and Chemical Engineering, Jiangsu Polytechnic University, Changzhou 213164, People’s Republic of China

## Abstract

The mol­ecule of the title compound, C_14_H_22_O_4_, is centrosymmetric. In the crystal, the mol­ecules are linked through O—H⋯O hydrogen bonds into a three-dimensional network.

## Related literature

For a related structure, see: Xi *et al.* (2008 ▶).
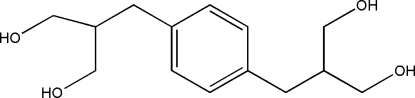


## Experimental

### 

#### Crystal data

C_14_H_22_O_4_
*M* *_r_* = 254.32
Orthorhombic, 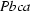

*a* = 9.939 (6) Å
*b* = 8.803 (5) Å
*c* = 15.366 (9) Å
*V* = 1344.5 (14) Å^3^
*Z* = 4
Mo *K*α radiation
μ = 0.09 mm^−1^
*T* = 291 (2) K
0.30 × 0.24 × 0.22 mm

#### Data collection

Bruker SMART APEX CCD diffractometer
Absorption correction: multi-scan (*SADABS*; Bruker, 2000 ▶) *T*_min_ = 0.97, *T*_max_ = 0.98
7571 measured reflections
1636 independent reflections
1215 reflections with *I* > 2σ(*I*)
*R*_int_ = 0.057

#### Refinement

*R*[*F* ^2^ > 2σ(*F* ^2^)] = 0.038
*wR*(*F* ^2^) = 0.115
*S* = 1.08
1636 reflections
88 parameters
2 restraints
H atoms treated by a mixture of independent and constrained refinement
Δρ_max_ = 0.19 e Å^−3^
Δρ_min_ = −0.17 e Å^−3^

### 

Data collection: *SMART* (Bruker, 2000 ▶); cell refinement: *SAINT* (Bruker, 2000 ▶); data reduction: *SAINT*; program(s) used to solve structure: *SHELXTL* (Sheldrick, 2008 ▶); program(s) used to refine structure: *SHELXTL*; molecular graphics: *SHELXTL*; software used to prepare material for publication: *SHELXTL*.

## Supplementary Material

Crystal structure: contains datablocks I, global. DOI: 10.1107/S1600536808041688/gk2172sup1.cif

Structure factors: contains datablocks I. DOI: 10.1107/S1600536808041688/gk2172Isup2.hkl

Additional supplementary materials:  crystallographic information; 3D view; checkCIF report

## Figures and Tables

**Table 1:** Hydrogen-bond geometry (Å, °)

*D*—H⋯*A*	*D*—H	H⋯*A*	*D*⋯*A*	*D*—H⋯*A*
O1—H1*A*⋯O2^i^	0.820 (16)	1.906 (17)	2.7254 (17)	177.4 (17)
O2—H2*A*⋯O1^ii^	0.820 (17)	1.943 (17)	2.7612 (17)	175.5 (17)

Symmetry codes: (i) 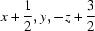; (ii) 
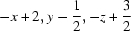
.

**Fig. 1.**
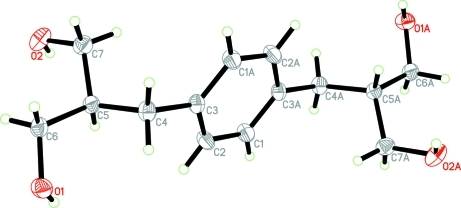
View of the title molecule showing the atom-labelling scheme; displacement ellipsoids are shown at the 30% probability level (symmetry code to generate atoms with the label A: 2-x, 1-y, 1-z)

**Fig. 2.**
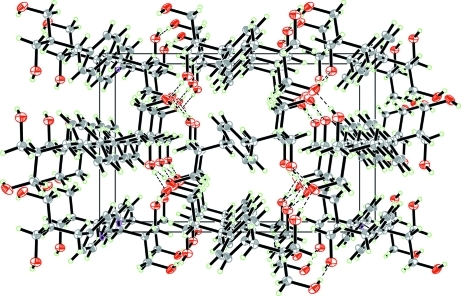
Crystal packing of the title compound viewed down the b direction. Dashed lines indicate hydrogen bonds.

## supplementary crystallographic information

### Comment

Reduction is a fundamental transformation in organic synthesis. Lithium aluminium hydride is used in organic synthesis as a powerful reducing agent.

The title molecule has a crystallographic inversion center located at the middle of the benzene ring. The *trans*-arrangement of two 1,3-dihydroxyisopropyl groups in the title compound was verified by X-ray crystallographic studies (Fig.1). The torsion angle C3—C4—C5—C6 is 165.14 (10) ° and the torsion angle of C3-C4-C5- C7 is -70.96 (13).

### Experimental

Tetraethyl 2,2'-(*p*-phenylenedimethylene)dimalonate was prepared according to the literature procedure (Xi *et al.*, 2008). In a flame-dryed, round-bottom flask was placed freshly distilled THF (80 ml) under dry nitrogen gas and the flask was placed in an ice-bath. Subsequently LiAlH_4_ (2.128 g, 56 mmol) was slowly added with stirring, followed by a dropwise addition of the solution of tetraethyl 2,2'-(*p*-phenylenedimethylene)dimalonate (2.95 g,7 mmol) in THF (20 ml) . After stirring for 3 h at room temperature, a saturated solution of Na_2_SO_4_(3 ml) was added. Stirring was continued for next 10 min. Then ethanol (8 mL) was added, the mixture was heated to 333 K. Lithium and aluminium salts were separated by filtration on celite. Filtrate was evaporated and the residue purified by crystallization, yielding the title compound (1.09 g, yield 61%; m.p. 448–449 K). Crystals suitable for X-ray analysis were obtained by slow evaporation of an aqueous solution at 288 K.

### Refinement

Carbon bound H atoms were placed geometrically and treated as riding on their carriers, with methylene C—H distance of 0.97 Å, aromatic C—H of 0.93 Å and *U*_iso_(H) = 1.2Ueq(C). H atoms from hydroxyl groups were refined with the distance restraint of O—H = 0.82 (2) Å and *U*_iso_(H) = 1.5 *U*_eq_(O)..

### Crystal data

**Table:**

C_14_H_22_O_4_	*D*_x_ = 1.256 Mg m^−^^3^
*M**_r_* = 254.32	Melting point = 448–449 K
Orthorhombic, *P**b**c**a*	Mo *K*α radiation, λ = 0.71073 Å
Hall symbol: -P 2ac 2ab	Cell parameters from 2317 reflections
*a* = 9.939 (6) Å	θ = 2.6–27.1°
*b* = 8.803 (5) Å	µ = 0.09 mm^−^^1^
*c* = 15.366 (9) Å	*T* = 291 K
*V* = 1344.5 (14) Å^3^	Block, colourless
*Z* = 4	0.30 × 0.24 × 0.22 mm
*F*(000) = 552	

### Data collection

**Table:**

Bruker SMART APEX CCD diffractometer	1636 independent reflections
Radiation source: sealed tube	1215 reflections with *I* > 2σ(*I*)
graphite	*R*_int_ = 0.057
φ and ω scans	θ_max_ = 28.3°, θ_min_ = 2.7°
Absorption correction: multi-scan (*SADABS*; Bruker, 2000)	*h* = −5→13
*T*_min_ = 0.97, *T*_max_ = 0.98	*k* = −11→11
7571 measured reflections	*l* = −20→20

### Refinement

**Table:**

Refinement on *F*^2^	Primary atom site location: structure-invariant direct methods
Least-squares matrix: full	Secondary atom site location: difference Fourier map
*R*[*F*^2^ > 2σ(*F*^2^)] = 0.038	Hydrogen site location: inferred from neighbouring sites
*wR*(*F*^2^) = 0.115	H atoms treated by a mixture of independent and constrained refinement
*S* = 1.08	*w* = 1/[σ^2^(*F*_o_^2^) + (0.06*P*)^2^] where *P* = (*F*_o_^2^ + 2*F*_c_^2^)/3
1636 reflections	(Δ/σ)_max_ < 0.001
88 parameters	Δρ_max_ = 0.19 e Å^−^^3^
2 restraints	Δρ_min_ = −0.17 e Å^−^^3^

### Special details

**Table:**

Geometry. All e.s.d.'s (except the e.s.d. in the dihedral angle between two l.s. planes) are estimated using the full covariance matrix. The cell e.s.d.'s are taken into account individually in the estimation of e.s.d.'s in distances, angles and torsion angles; correlations between e.s.d.'s in cell parameters are only used when they are defined by crystal symmetry. An approximate (isotropic) treatment of cell e.s.d.'s is used for estimating e.s.d.'s involving l.s. planes.
Refinement. Refinement of *F*^2^ against ALL reflections. The weighted *R*-factor *wR* and goodness of fit *S* are based on *F*^2^, conventional *R*-factors *R* are based on *F*, with *F* set to zero for negative *F*^2^. The threshold expression of *F*^2^ > σ(*F*^2^) is used only for calculating *R*-factors(gt) *etc*. and is not relevant to the choice of reflections for refinement. *R*-factors based on *F*^2^ are statistically about twice as large as those based on *F*, and *R*- factors based on ALL data will be even larger.

### Fractional atomic coordinates and isotropic or equivalent isotropic displacement parameters (Å^2^)

**Table:**

	*x*	*y*	*z*	*U*_iso_*/*U*_eq_	
C1	1.09492 (13)	0.40412 (13)	0.53366 (8)	0.0388 (3)	
H1	1.1600	0.3402	0.5572	0.047*	
C2	1.09178 (14)	0.55517 (13)	0.55850 (8)	0.0396 (3)	
H2	1.1548	0.5910	0.5982	0.048*	
C3	0.99643 (11)	0.65355 (12)	0.52522 (8)	0.0316 (3)	
C4	0.99230 (13)	0.81711 (12)	0.55423 (8)	0.0347 (3)	
H4A	0.9288	0.8719	0.5180	0.042*	
H4B	1.0804	0.8620	0.5454	0.042*	
C5	0.95220 (12)	0.83713 (12)	0.64958 (7)	0.0303 (3)	
H5	1.0045	0.7646	0.6842	0.036*	
C6	0.98372 (12)	0.99558 (14)	0.68286 (9)	0.0371 (3)	
H6A	0.9369	1.0694	0.6471	0.045*	
H6B	0.9502	1.0053	0.7419	0.045*	
C7	0.80474 (13)	0.80040 (14)	0.66200 (8)	0.0399 (3)	
H7A	0.7512	0.8777	0.6333	0.048*	
H7B	0.7853	0.7039	0.6342	0.048*	
O1	1.12348 (9)	1.02918 (10)	0.68214 (6)	0.0417 (3)	
H1A	1.1656 (18)	0.9562 (18)	0.7010 (10)	0.063*	
O2	0.76614 (10)	0.79212 (10)	0.75078 (6)	0.0471 (3)	
H2A	0.8000 (18)	0.7167 (18)	0.7731 (12)	0.071*	

### Atomic displacement parameters (Å^2^)

**Table:**

	*U*^11^	*U*^22^	*U*^33^	*U*^12^	*U*^13^	*U*^23^
C1	0.0425 (7)	0.0340 (7)	0.0399 (7)	0.0101 (5)	−0.0108 (5)	−0.0035 (5)
C2	0.0430 (7)	0.0361 (7)	0.0398 (7)	0.0027 (5)	−0.0128 (6)	−0.0076 (5)
C3	0.0383 (7)	0.0269 (6)	0.0297 (6)	0.0000 (5)	0.0018 (5)	−0.0016 (4)
C4	0.0434 (7)	0.0249 (6)	0.0357 (7)	−0.0014 (5)	0.0023 (5)	0.0003 (5)
C5	0.0335 (6)	0.0231 (5)	0.0343 (6)	0.0006 (4)	0.0004 (5)	−0.0012 (4)
C6	0.0384 (7)	0.0287 (6)	0.0443 (8)	0.0004 (5)	0.0028 (6)	−0.0077 (5)
C7	0.0375 (7)	0.0363 (6)	0.0460 (7)	−0.0018 (5)	0.0032 (6)	−0.0002 (5)
O1	0.0390 (5)	0.0296 (5)	0.0564 (6)	−0.0040 (4)	−0.0023 (4)	−0.0028 (4)
O2	0.0485 (6)	0.0379 (5)	0.0550 (6)	0.0090 (4)	0.0188 (5)	0.0089 (4)

### Geometric parameters (Å, °)

**Table:**

C1—C3^i^	1.3787 (17)	C5—C6	1.5183 (17)
C1—C2	1.3838 (17)	C5—H5	0.9800
C1—H1	0.9300	C6—O1	1.4203 (18)
C2—C3	1.3819 (17)	C6—H6A	0.9700
C2—H2	0.9300	C6—H6B	0.9700
C3—C4	1.5078 (18)	C7—O2	1.4190 (18)
C4—C5	1.5285 (18)	C7—H7A	0.9700
C4—H4A	0.9700	C7—H7B	0.9700
C4—H4B	0.9700	O1—H1A	0.820 (16)
C5—C7	1.513 (2)	O2—H2A	0.820 (17)
			
C3^i^—C1—C2	121.33 (11)	C7—C5—H5	107.8
C3^i^—C1—H1	119.3	C6—C5—H5	107.8
C2—C1—H1	119.3	C4—C5—H5	107.8
C3—C2—C1	121.04 (12)	O1—C6—C5	112.98 (10)
C3—C2—H2	119.5	O1—C6—H6A	109.0
C1—C2—H2	119.5	C5—C6—H6A	109.0
C1^i^—C3—C2	117.63 (11)	O1—C6—H6B	109.0
C1^i^—C3—C4	121.85 (11)	C5—C6—H6B	109.0
C2—C3—C4	120.51 (11)	H6A—C6—H6B	107.8
C3—C4—C5	113.62 (10)	O2—C7—C5	113.21 (11)
C3—C4—H4A	108.8	O2—C7—H7A	108.9
C5—C4—H4A	108.8	C5—C7—H7A	108.9
C3—C4—H4B	108.8	O2—C7—H7B	108.9
C5—C4—H4B	108.8	C5—C7—H7B	108.9
H4A—C4—H4B	107.7	H7A—C7—H7B	107.7
C7—C5—C6	110.72 (10)	C6—O1—H1A	109.5 (12)
C7—C5—C4	110.41 (10)	C7—O2—H2A	109.5 (12)
C6—C5—C4	112.03 (10)		
			
C3^i^—C1—C2—C3	0.2 (2)	C3—C4—C5—C6	165.14 (10)
C1—C2—C3—C1^i^	−0.2 (2)	C7—C5—C6—O1	173.08 (10)
C1—C2—C3—C4	178.72 (12)	C4—C5—C6—O1	−63.19 (14)
C1^i^—C3—C4—C5	111.75 (15)	C6—C5—C7—O2	−65.22 (13)
C2—C3—C4—C5	−67.11 (15)	C4—C5—C7—O2	170.13 (9)
C3—C4—C5—C7	−70.96 (13)		

Symmetry codes: (i) −*x*+2, −*y*+1, −*z*+1.

### Hydrogen-bond geometry (Å, °)

**Table:**

*D*—H···*A*	*D*—H	H···*A*	*D*···*A*	*D*—H···*A*
O1—H1A···O2^ii^	0.820 (16)	1.906 (17)	2.7254 (17)	177.4 (17)
O2—H2A···O1^iii^	0.820 (17)	1.943 (17)	2.7612 (17)	175.5 (17)

Symmetry codes: (ii) *x*+1/2, *y*, −*z*+3/2; (iii) −*x*+2, *y*−1/2, −*z*+3/2.
